# Development, Fabrication, and Characterization of Composite Polycaprolactone Membranes Reinforced with TiO_2_ Nanoparticles

**DOI:** 10.3390/polym11121955

**Published:** 2019-11-28

**Authors:** Karina del Ángel-Sánchez, César I. Borbolla-Torres, Luis M. Palacios-Pineda, Nicolás A. Ulloa-Castillo, Alex Elías-Zúñiga

**Affiliations:** 1 Departamento de Ingeniería Mecánica y Materiales Avanzados, Tecnologico de Monterrey, School of Engineering and Science. Av. E. Garza Sada 2501 Sur, Monterrey, NL 64849, Mexico; karina.delangels@gmail.com (K.d.Á.-S.); cesar.ivan.borbolla@gmail.com (C.I.B.-T.); nicolas.ulloa@tec.mx (N.A.U.-C.); 2 Tecnológico Nacional de México/Instituto Tecnológico de Pachuca, Pachuca, Hidalgo 42082, Mexico; palacios@itpachuca.edu.mx

**Keywords:** composite membranes, TiO_2_ nanoparticles, chloroform and *N,N*-dimethylformamide (DMF) solvent, polycaprolactone, hydrophobicity

## Abstract

This paper focuses on developing, fabricating, and characterizing composite polycaprolactone (PCL) membranes reinforced with titanium dioxide nanoparticles (NPs) elaborated by using two solvents; acetic acid and a mixture of chloroform and N,N-dimethylformamide (DMF). The resulting physical, chemical, and mechanical properties of the composite materials are studied by using experimental characterization techniques such as scanning electron microscopy (SEM), differential scanning calorimetry (DSC), X-ray diffraction (XRD), Fourier-transform infrared (FTIR) analysis, contact angle (CA), uniaxial and biaxial tensile tests, and surface roughness measurements. Experimental results show that the composite material synthesized by sol-gel and chloroform-DMF has a better performance than the one obtained by using acetic acid as a solvent.

## 1. Introduction

Polycaprolactone (PCL) is a well-known semi-crystalline and hydrophobic polymer that degrades at a slower rate than other biocompatible polymers such as polyglycolide (PGA) and poly (d,l-lactide) (PDLA), to name a few. PCL has superior rheological and viscoelastic properties that facilitate its manufacturability to produce biocompatible devices, from sutures and wound dressings to bone engineering artifacts [1,2,3]. Its biodegradability properties widen its commercial applications, not only in the biomedical field, but also in plastics production [4]. Furthermore, the addition of carbon nanotubes (CNT), graphene, and other nanostructures expand the usage of PCL for the fabrication of flexible supercapacitors [5,6]. Recently, the compostable properties of PCL blend with polylactic acid (PLA), polyhydroxyoctanoate (PHO), or thermoplastic starch (TPS) have been investigated. It was fond that PCL-TPS (70/30) and PCL-PHO (85/15) achieve the biodegradable bench mark in the soil test environment, and the blend PLA-PCL (80/20) is feasible for a home compostability blend [7]. As a healing reagent, PCL has self-healing capacity under thermal initiation, and influences the composite material shape memory capacity. In fact, PCL can be used to make more durable and efficient flexible shape memory-based supercapacitors when mixed with shape memory polyurethane (SMPU), carbon nanotubes (CNT), and manganese dioxide (MnO2). The addition of PCL improves self-healing effects and also triggers shape recovery by promoting surface voids to heal [6,8]. Furthermore, PCL has been reinforced with TiO_2_ (pure anatase and rutile) nanoparticles to produce composite fibers by the electrospinning method for enhancing mechanical properties, cell proliferation, and cell adhesion [9], and for the development of wound dressings [10]. It has also been found that PCL/TiO_2_ nanoparticles decrease water absorption and swelling ratio and tend to improve the compressive strength properties needed to fabricate bone scaffolds [11]. Depending on the PCL application, it is possible to accelerate its degradation by increasing aging temperature, which results in an approximate two-fold increase in PCL degradation rate, or by using sodium hydroxide (NaOH) to enhance surface erosion that accelerates the degradation process in a more homogeneous manner [12].

To further advanced the advantages that the PCL-TiO_2_ blend has, this paper focuses on studying the physicochemical properties of novel PCL composite membranes elaborated by using acetic acid and chloroform-*N,N*-dimethylformamide (DMF) solvents. The structural composite dependence of the developed materials will be investigated when P25 Degussa and TiO_2_ nanoparticles, synthetized by a sol-gel method, are used. The effect of both solvents during PCL dissolution will be explored by scanning electron microscopy (SEM) and infinite focus microscope (Alicona) to observe differences between the surface roughness conditions of the composite membranes. Structural properties will be investigated by differential scanning calorimetry (DSC) and X-ray diffraction (XRD) to detect structural modifications in the polymeric semi-crystalline domains when TiO_2_ nanoparticles are used. The effective interaction of the TiO_2_ nanoparticles and PCL will be explored by Fourier-transform infrared spectroscopy (FTIR) analyses. The produced composite membrane’s permeability behavior will be studied by contact angle (CA) measurements. Finally, the mechanical performance of the developed composite material samples will be addressed by performing uniaxial and equibiaxial tests, and surface roughness measurements.

## 2. Materials and Methods 

### 2.1. Materials

The reactived materials used for the synthesis of TiO_2_ and for the composite membranes are deionized water, nitric acid (95%), titanium (IV) butoxide (97%), P25 Degussa PCL (M_W_ 80,000), chloroform, DMF, and acetic acid (95%). All the materials were purchased from Sigma–Aldrich (México City, México) and used without any further purification. 

### 2.2. TiO_2_ Synthesis by Sol-gel Method

TiO_2_ nanoparticle synthesis was carried out by mixing deionized water, nitric acid, and titanium (IV) butoxide in a 3-mouth flask at 25 °C under magnetic agitation. Subsequently, the solution was heated to 70–90 °C for 8–12 h and assisted with a water cooler reflux. The gel obtained was dried at 80 °C for 10 h and thermally annealed at 400 °C for 4 h to remove organic material, solvents, and humidity. The procedure of the synthesis is explained in [13].

### 2.3. PCL Composite Membranes Manufacturing Process

The fabrication of the membranes was performed using two solvents; a mixture of chloroform-DMF in a ratio of 97:3 (V:V) and acetic acid. The procedure consisted of diluting PCL in the solvents to obtain a 10% polymeric matrix solution. Subsequently, synthesized TiO_2_ and P25 Degussa nanoparticles (NPs) were added in different concentrations to obtain the doped ones. The concentrations for the solutions used are listed in Table 1. The polymeric solution was then homogeneously deposited on a glass substrate and dried at room temperature for 8 h. Afterwards, each membrane underwent a washing process, which consisted of three cycles of 5 min for each membrane surface under the water faucet, and 1 min of gentle shaking between cycles.

### 2.4. Differential Scanning Calorimetry (DSC)

The degree of crystallinity and the melting temperature of PCL were measured using DSC Perkin–Elmer Pyris 8000 equipment (México City, México). The melting temperature was determined considering the onset melting endotherm ($T_{m}^{onset}$). The degree of crystallinity ($\chi_{c}$) was estimated using the enthalpy of melting change according to the equation: $\chi_{c}=100\cdot \Delta H_{m}/\left\{\Delta H_{m}^{0}\left(1-W_{f}\right)\right\}$, where $\Delta H_{m}$ and $\Delta H_{m}^{0}$ are the melting and the fully crystalline melting (139.5 J/g) enthalpies of the PCL, respectively, and $W_{f}$ is the dopant weight fraction. All the samples (average weight 10 mg) were held in standard aluminum pans and covers. The specimens were scanned from 50 °C to 70 °C, with a heating rate of 10 °C/min in a nitrogen gas atmosphere. The DSC analysis curves for the material samples was carried out for the materials as received (first run data).

### 2.5. Thermogravimetric Analysis (TGA)

The thermogravimetric analyses were performed using TGA Perkin–Elmer Pyris 8000 equipment (México City, México) with a heating rate of 10 °C/min. The samples were scanned in a temperature range from 50 °C to 700 °C using air as a purge gas.

### 2.6. X-ray Diffraction (XRD)

XRD measurements of the developed composite membranes and bare materials were carried out using a PanAnalytical X’Pert Pro PW1800 diffractometer with a scanning rate of 2 °/min and by using Cu–Kα radiation (México City, México). The system was operated at 45 mA and 40 kV, and the XRD data was collected in the 2θ range of 10°–40°.

### 2.7. Scanning Electron Microscopy (SEM)

A SEM (ZEISS model EVO MA 25) was used to investigate the morphology of the composite membranes. The equipment was operated with an accelerating voltage of 10.00 kV and a work distance of 7.0 mm.

### 2.8. Fourier-Transform Infrared (FTIR) Analysis

FTIR (Perkin–Elmer Frontier, México City, México) equipment with a universal attenuated total reflectance (UATR) polarization accessory was used to carry out the IR analysis. The procedure consisted of placing the developed composite membranes on the ZnSe-diamond crystal of the UATR and pressing it with a tip to assure a good contact between the sample and the incident IR beam. The IR spectra were measured in the interval range of 4000 cm^−1^ to 400 cm^−1^ with a resolution of 4 cm^−1^, and by considering an average of 16 scans. 

### 2.9. Contact Angle (CA) Test 

CA measurements were performed using a Data physics (OCA, 15EC) system. The CA was measured between the composite membranes surface and a water droplet using SCA20_U software (México City, México). The procedure consisted of placing a water droplet from a dispenser, with a volume capacity of 10 μL and deposition ratio of 2 μL/s, onto the composite membrane’s surface. The values retrieved from the CA characterization corresponded to 10 measurements per sample, taking into account the averages readings obtained between the droplet’s right and left side for each measurement. 

### 2.10. Uniaxial and Biaxial Tensile Tests

Uniaxial tests were performed using Instron 3365 equipment with a cell load capacity between 5–5000 N. The calculations of the maximum tensile strength were retrieved by considering the average value recorded from four measurements according to the norm ISO-527-3 specimen type 4. Biaxial cyclic loading tests were performed on three specimens of the same material samples using an ElectroForce LM1 TestBench (México City, México). The major components of this system consist of four linear actuators and load cells attached to the end of the actuators, one in each direction. The maximum load allowed in the actuators is 17 N, with maximum displacement of 19 mm. The strain field values on the sample surface were measured using a digital image correlation (DIC) system, Aramis V8. The measurements were performed at room temperature using a cruciform sample geometry [14]. The corresponding experimental tests were carried out by considering three loading and unloading cycles with specified displacement of 1, 2, and 3 mm.

In order to study the biaxial response caused by the loading and unloading cycles and the Mullins effect, the following expression, proposed in [14,15],

(1) $$\begin{array}{ll} \tau_{j}-\tau_{k}= & \{\left[\left(1-f\right)ℵ+\frac{2f}{3}\left(A_{1}+\frac{2A_{2}}{3}\left(I_{1i}-3\right)\right)\right]\left(\lambda_{j}^{2}-\lambda_{k}^{2}\right)+\frac{G}{2c}[\lambda_{j}f_{i}\left(\lambda_{1},\lambda_{2},\;\lambda_{3}\right)-\\ & \lambda_{k}f_{k}\left(\lambda_{1},\lambda_{2},\;\lambda_{3}\right)]\}e^{-b\sqrt{\frac{m}{M}\left(M-m\right)}},\;j\;\neq \;k,\;1,\;2,\;3\;\left(no\;sum\right). \end{array}$$

is used to describe the Cauchy stress-softening material behavior. Here, *c* is a constant parameter related to residual strains, *b* is a dimensionless material softening parameter, *m* represents the stretch intensity, which is defined for equibiaxial extension as $m=\sqrt{2\lambda^{2}+\lambda^{-8}}$, $m_{max}=M$ is the amount of maximum strain intensity at the point at which the material is unloaded, ℵ is a material response function, and λ represents the sample stretch. The corresponding principal engineering stress, σ, can be computed from the expression $\sigma =TF^{-1}$ where $F^{-1}$ represent the inverse of the tensor deformation gradient and *T* is the usual Cauchy stress tensor [15].

### 2.11. Surface Roughness Measurements

An Alicona Infinite Focus microscope (México City, México) was used to determine the roughness of the composite membranes. The parameters used to analyze the surface condition of the membranes were 700 μs of brightness, a contrast of 4 with an objective overview of 20X, vertical resolution of 0.60 μm, and lateral resolution of 6 μm. The roughness average values were obtained according to the norm ISO-4287.

## 3. Results and Discussion

### 3.1. Morphology of PCL Membrane 

**Figure 1:**
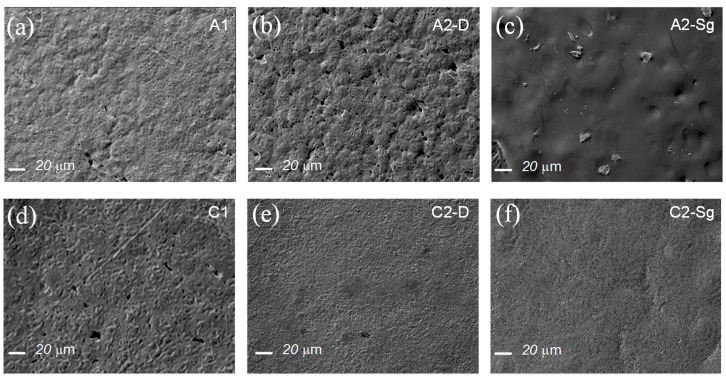
Surface SEM images of the composite membranes: (**a**) Sample A1. PCL diluted in acetic acid. Notice that because of the solvating property of the acetic acid solvent, the PCL composite surface structure shows the appearance of micro-mesopores. (**b**) Sample A2-D. PCL-P25 compound increases surface porosity because of the affinity of the acetic acid. (**c**) Sample A2-Sg. The TiO_2_ NPs were annealed at 400 °C for 4 h to remove organic materials, solvents, and humidity. Because of the affinity between the annealed TiO_2_ NPs with PCL and acetic acid, the resulting surface structure exhibits low porosity and some pinholes. (**d**) Sample C1. PCL diluted in chloroform-DMF solvent. The non-solvating chloroform-DMF porogen solvent has good miscibility with PCL and, thus, the elaborated material promotes more uniform membrane pore structure resulting in the formation of macropores, small surface, area and greater pore volume. (**e**) Sample C2-D. PCL-P25 diluted in chloroform-DMF solvent. Notice that the presence of the P25 titanium-dioxide NPs in the bulk mixture generates a compound with scarce porosity. (**f**) Sample C2-Sg. The TiO_2_ NPs were annealed at 400 °C for 4 h to remove organic materials, solvents, and humidity. Because of the affinity of TiO_2_ NPs and the solubility of chloroform-DMF solvent with PCL, the resulting surface structure exhibits smaller pore volume.

Figure 1 illustrates the composite membranes manufactured with solvating acetic acid and non-solvating chloroform-DMF. Because of the solvent physical properties, listed in Table 2, PCL diluted in acetic acid and reinforced with TiO_2_ NPs leads to a greater surface area and the appearance of micro pores, as illustrated in Figure 1a,b,c, while the non-solvating chloroform porogenic solvent generates membrane macropores (see Figure 1d,e,f). Based on the solvent physical properties in Table 2 [16,17], chloroform and DMF have a similar boiling point with low solubility parameter differences and exhibits good solubility and miscibility with PCL polymeric material. Therefore, the PCL composite membranes elaborated with this solvent promote more uniform macro pore structure [18,19,20,21]. Also, it should be noted that when TiO_2_ NPs are added to the PCL matrix, the nanoparticle concentration, affinity, compatibility, and interaction with the polymer matrix and solvents affect the appearance of the porous-like structure in the produced composite membranes, which influences their resulting properties, such as strength, ductility, surface roughness, and contact angle, among others. 

### 3.2. Thermogravimetry Analysis

**Figure 2:**
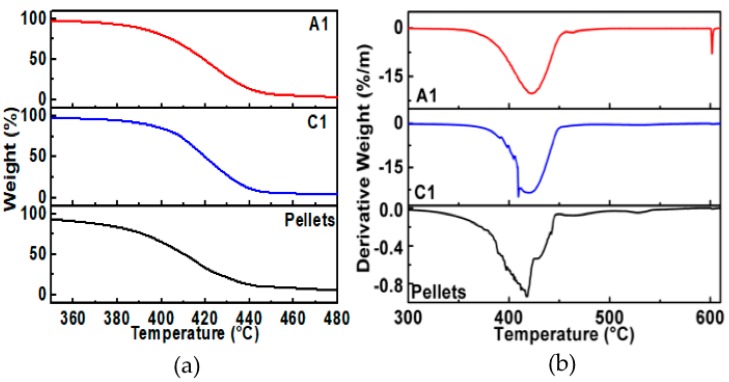
Thermogravimetric analysis (TGA) thermal degradation of the PCL composite membranes using acetic acid (A1) and a combination of chloroform and DMF (C1) solvents. (**a**) Thermal degradation and (**b**) derivate of the decomposition.

Thermal degradation analyses for PCL pellets (the reference or bare material) and developed A1 and C1 composite membranes are shown in Figure 2. Temperature dependence of the PCL reference and samples A1 and C1 shows one-step weight loss between 360–450 °C, with an inflection point around 415 °C. Such thermal behavior is attributed to the PCL decomposition of methyl pentanoate, water, and carbon dioxide [22].

### 3.3. Differential Scanning Calorimetry (DSC) 

**Figure 3:**
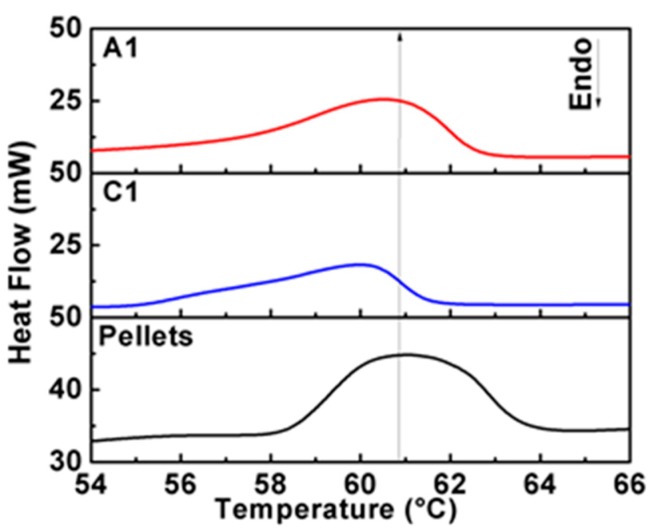
Differential scanning calorimetry (DSC) thermograms of the PCL membranes using acetic acid (A1) and a combination of chloroform and DMF (C1) as solvent.

In order to investigate the structural modifications in PCL caused by the solvents, DSC thermograms were obtained for samples A1 and C1 and were compared to those of the PCL reference material. As illustrated in Figure 3, the thermogram of the PCL reference material shows an onset of melting temperature at 58.8 °C, as well as a broader melting curve between 58 °C and 64 °C. The thermogram for sample A1 (PCL membrane dissolved in acetic acid) shows a similar behavior than the PCL reference material, with a decrement of its amplitude and widening of the thermogram curves. These changes suggest that the acetic acid modifies, in a low proportion, the PCL crystal domains. On the other hand, the thermogram for sample C1 (PCL membrane dissolved in chloroform-DMF) shows a shift to lower temperatures (below 60 °C) with a broader temperature interval, like the one illustrated in sample A1.

The values calculated for,$\;T_{m}^{onset}$, $\Delta H_{m}^{0}$, and $\chi_{c}$ are listed in Table 3. The $\chi_{c}$ value calculated for the PCL reference material of 39.2 % is lower than the one calculated at 53.6% for the sample A1. Also, notice that the value of $\chi_{c}=30.3\%$ for the sample C1 is lower than that of sample A1. The increment in the degree of crystallinity value in sample A1 is due to the broadening of its DSC curve. However, for sample C1, the decrement in its degree of crystallinity value indicates that the solvent has an impact in the formation of the PCL crystal domains, as is observed by the DSC measurements. SEM results in combination with DSC suggest that the chloroform-DMF promotes a better interaction with the PCL polymeric chains.

### 3.4. X-ray Diffraction (XRD)

**Figure 4:**
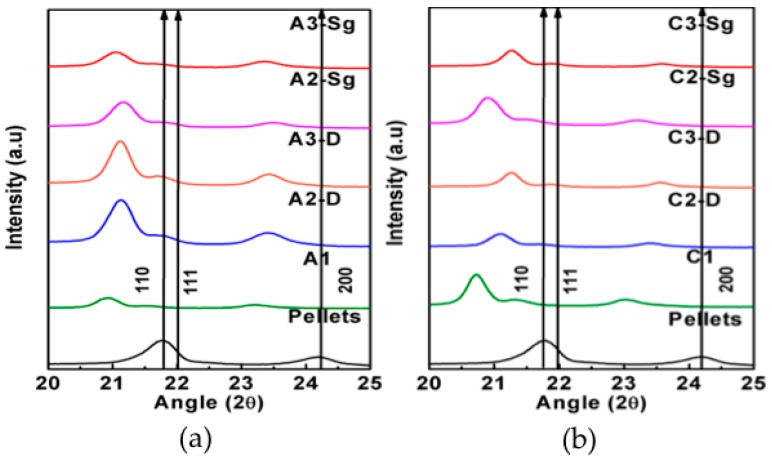
XRD diffractograms of the PCL composite membranes manufactured using (**a**) acetic acid and (**b**) chloroform-DMF solvent.

The diffractogram for the PCL reference material shows two peaks at 21.7° and 24.1°, corresponding to (110) and (200) diffraction planes, as shown in Figure 4. These peaks are characteristic of the PCL orthorhombic unit cell [23]. As expected, the use of acetic acid solvent during the preparation of the PCL membrane (A1), modifies the formation of crystal domains because of the shift shown in Figure 4a to lower angles of the (110) and (200) planes, as well as the detection of the (111) plane. The same behavior was detected when the TiO_2_ was incorporated in the composites A2-D, A3-D, A2-Sg, and A3-Sg, which indicates that the NPs do not play an important role during the PCL crystallization, thus proving that the main change is induced by the solvents.

The diffractogram for the PCL membrane using chloroform-DMF (C1) shows the same behavior observed in A1 and, contrary to the diffractograms for composite membranes produced with acetic acid, the samples fabricated with chloroform-DMF solvents, C2-D, C3-D, C2-Sg, and C3-Sg, show different shifts and crystallizations. Also notice from Figure 4 that the crystallization rate of PCL is changed by adding TiO_2_ NPs and the solvent used to dilute the polymeric matrix, as observed by the intensities for a given band [24]. These intensities revealed that the solvent and the addition of TiO_2_ NPs influences the PCL composite membranes crystallization. It is also observed that the compound crystallization changes with the increasing content of TiO_2_ NPs. Thus, the concentration of TiO_2_ NPs and the solvent physical properties influences the PCL membrane pore structure as well as its physical properties [25,26], as will be discussed later on. 

### 3.5. Fourier-Transform Infra Red (FTIR)

**Figure 5:**
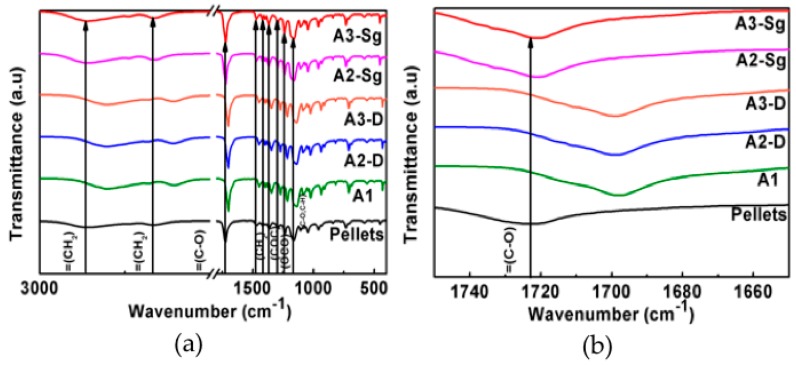
(**a**) FT-spectra shows wavenumber shifts between the material samples A1, A2-D, and A3-D and the Pellets. (**b**) Samples spectra zoom in the lower wave number values.

**Figure 6:**
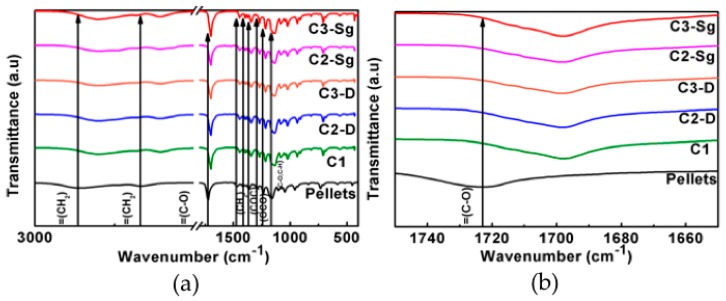
(**a**) FTIR spectra shows wavenumber shifts between the material samples C1, C2-D, and C3-D and the Pellets. (**b**) Samples spectra zoom in at lower wave number values.

The IR analyses of the developed composite membranes exhibit the characteristic vibrational modes of the PCL, as shown in Figure 5 and Figure 6. The FTIR spectrum measured for the PCL reference material shows vibration modes at 2925 cm^−1^ and 2840 cm^−1^, which corresponds to asymmetric (*ν*_as_) and symmetric (*ν*_s_) stretching modes originated by CH=O groups and strong bands around 1725 cm^−1^ and 1698 cm^−1^, which are related to the C=O stretching (*ν*_s_) mode. The presence of CH_2_ and CH_3_ groups, related to bending (δ) modes, correspond to the range 1473 cm^−1^ to 1342 cm^−1^ bonds, respectively. Also, the regions of 1294 cm^−1^ and 1157 cm^−1^ are associated with the crystalline (*ν*_cr_) and amorphous (*ν*_am_) characteristics that correspond to the C–O and C–C modes. On the other hand, the modes detected at 1240 cm^−1^ and 1170 cm^−1^ are associated with the asymmetric (*ν*_as_) and symmetric (*ν*_s_) modes originated by C–O–C groups. Figure 5 shows the FTIR spectra of the composite membranes developed by using acetic acid. Based on the recorded data, one can see from Figure 5 that only for the material samples A1, A2-D, and A3-D does the band for the C–O mode exhibit a shift to lower wavenumbers when compare to the pellets spectra, due to the interaction between the solvent and TiO_2_ NPs, as well as the thermal stability material enhancement [27]. This wavenumber shift is not occurring for the samples A2-Sg and A3-Sg. Similarly, the same shifts to lower wave numbers for the composite membranes C1, C2-D, and C3-D were recorded during experimental measurements, as illustrated in Figure 6. This shift is an indication of the compatibility between the reagents used. 

### 3.6. Contact Angle Tests

**Figure 7:**
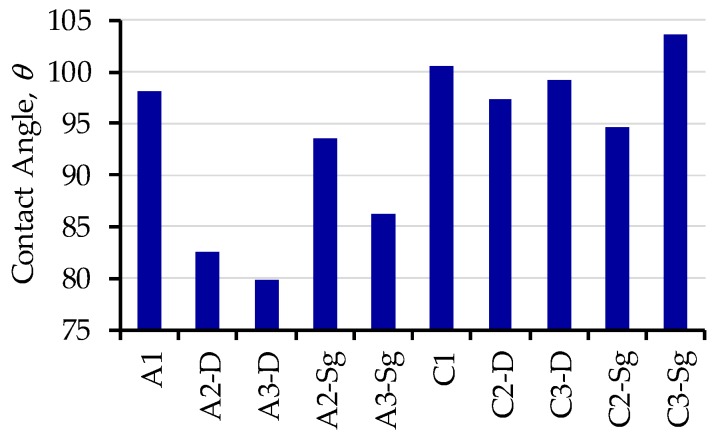
Contact angle (CA) collected from the developed composite membranes using P25 Degussa and TiO2-Sg nanoparticles.

Experimental values collected from contact angle (CA) and uniaxial extension tests are displayed in Figure 7. From CA tests, one can see from Figure 7 that the reference samples A1 and C1 exhibit a similar hydrophobic permeability behavior due to the presence of CH_2_ groups in the PCL backbone. For composite membrane samples elaborated with acetic acid (A2-D, A3-D, A2-Sg, A3-Sg), a relative decrement in the contact angle is observed in Figure 7 because of the reagents low compatibility. Also, notice from Figure 7 that the samples C2-D, C3-D, C2-Sg, and C3-Sg exhibit slight changes on their CA, while maintaining a hydrophobic behavior. In this case, sample C3-Sg shows the highest CA value. 

### 3.7. Uniaxial Extension Tests

**Figure 8:**
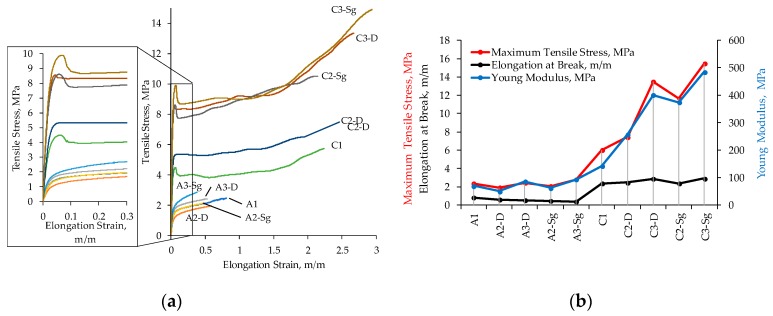
Simple uniaxial extension tests performed on the produced membrane composite samples. (**a**) Stress-strain response behavior curves. (**b**) Maximum tensile, elongation at break, and Young moduli values obtained for the different composite samples. Notice that the PCL samples diluted in chloroform-DMF solvent show the typical glassy-polymer elastic-plastic material response behavior; linear behavior, non-linear response transition to global yield, followed by stress-softening and, for increasing strain values, by hardening effects [28,29]. It is also seen that each of these material behavior features are exhibited for all the membrane compounds in which chloroform-DMF solvent was used. However, for those membrane compounds in which PCL was diluted in acetic acid solvent, the material response behavior exhibits linear behavior and a subsequent nonlinear response before samples failure. The back stress and hardening effects were not detected during experimental tests because the use of acetic acid as a solvent deteriorates PCL chain ductility. The variation in the maximum tensile stress value is mainly due to the interaction of the polymer matrix with the TiO_2_ NPs. The maximum strength value reduction is due to the solvating and affinity properties that the acetic acid solvent has with PCL, which enhances the appearance of micro-mesopores that hinders the resulting compound mechanical properties.

Experimental data obtained from uniaxial test measurements are illustrated in Figure 8. Notice from Figure 8 that the maximum tensile strengths of PCL composite membranes increased with the TiO2 NPs and the type of solvent. In fact, sample C3-Sg, that is a PCL compound with TiO2 NPs annealed at 400 °C diluted in chloroform-DMF solvent, exhibits the maximum tensile strength value, which is 418% higher than those of the same compound, A3-Sg, but diluted in acetic acid. Figure 8 shows that for all material compounds diluted in chloroform-DMF solvent, the elongation strain at break increased, while for those compounds diluted with acetic acid, the amount of elongation strain at break decreased, indicating that the incorporation and concentration of TiO_2_ influences the compound mechanical performance. Therefore, and based on the strength values plotted in Figure 8 and listed in Table 4, it is concluded that the mechanical properties of the composite membranes are highly dependent on the polymer-nanoparticle interface properties, molarity of TiO2 NPs, and on the solvent used to dilute the material mixture. In fact, the incorporation of TiO_2_ NPs and chloroform-DMF solvent enhances the ductility of the PCL chains because of the good solvent miscibility, reduction of the membrane porosity surface, and good interfacial adhesion between the polymer matrix and the TiO_2_ NPs. In contrast, the usage of acetic acid as a solvent deteriorated the ductility of the PCL chains, but the interaction of TiO_2_ NPs slightly increased the maximum tensile strength, which is, in general for the different produced membranes with acetic acid solvent, much lower than those fabricated with chloroform-DMF solvent. This reduction in maximum strength value is also due to the solvating and affinity properties that the acetic acid solvent has with PCL that enhances the appearance of micro-mesopores, as illustrated in Figure 1. In other words, due to the solvent physical properties listed in Table 2, the composite PCL membranes were not only influenced by the PCL/TiO_2_ NPs interface properties, but also by the usage of the solvent solution to produce the mixture.

### 3.8. Cyclic Biaxial Tests

**Figure 9:**
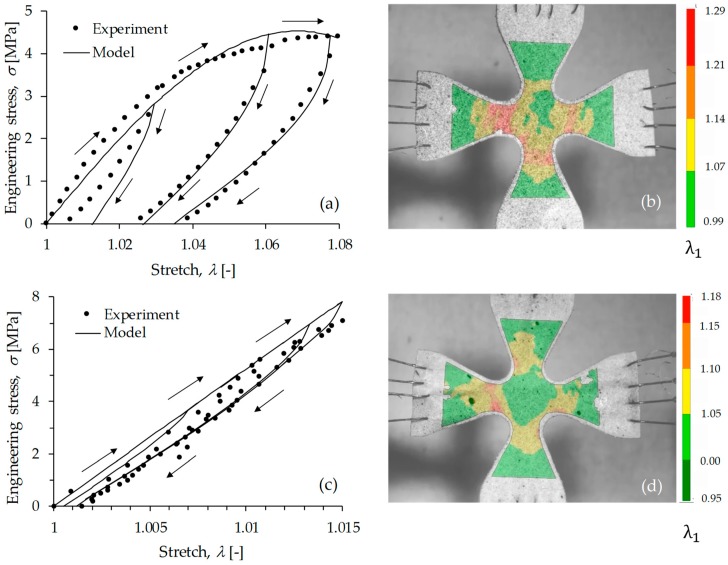
Equibiaxial loading-unloading cyclic tests for A1 and C1 material samples. (**a**) Sample A1 stress-stretch curve that corresponds to the central point of the cruciform specimen shown in (**b**). (**c**) Sample C1 stress-stretch curve that corresponds to the central point of the cruciform specimen shown in (**d**). Figures (**b**,**d**) illustrate experimental stretch measurement performed through digital image correlation. Experimental measurements are indicated by black dots, while the solid lines represent the computed theoretical predictions obtained from Equation (1). The arrows indicate the direction of loading and unloading cycles performed in the test.

Figure 9 shows the mechanical performance of samples A1 and C1 when subjected to equibiaxial cyclic tests. Experimental data are represented by black dots, while the solid lines describe theoretical predictions computed from Equation (1). The material shear modulus, *µ*, the chain number of links, *N*, the energy density parameters, *A*_1_ and *A*_2_, and the stress softening and residual stress parameter, *b* and *c*, used to fit experimental data in Equation (1), are summarized in Table 5. Notice that the shear modulus of sample C1 is higher than that of sample A1. Therefore, and based on the estimated parameter values of *b* and *c* listed in Table 5, one can conclude that there is a difference in the energy absorption capacity of both materials. Also, it is seen from Figure 9 that sample C1 exhibits lower material stretch permanent set values when compared to sample A1 under cyclic loading conditions.

### 3.9. Roughness Measurements 

**Figure 10:**
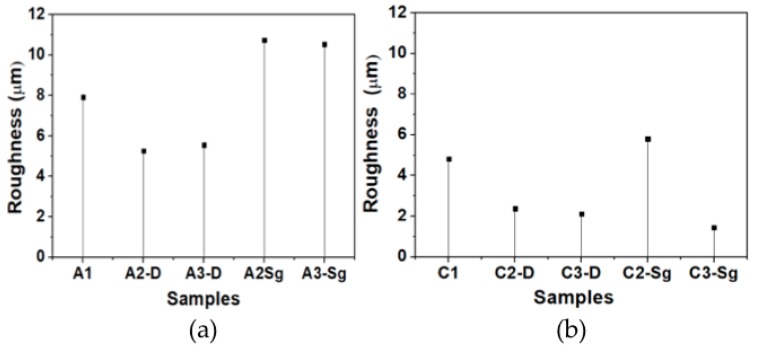
Membrane surface roughness values collected using infinity focus microscopy. (**a**) Samples made from PCL, P25 Degussa, TiO_2_ nanoparticles, and acetic acid as solvent. (**b**) Samples made from PCL, TiO_2_-Sg nanoparticles, and chloroform-DMF.

The average roughness values were obtained by focus variation microscopy technology (Alicona Infinite Focus microscope), following the ISO 4288 norm. In all the samples, measurements were performed in the lateral side (z-x plane) of the lattice structure with a filter waviness of *L*c = 80 μm and a profile length of *L* = 450 μm. The PCL membrane samples produced with acetic acid as a solvent were measured by considering the following parameters: 700 μs of brightness, a contrast of 4, objective overview of 20X, vertical resolution of 0.60 μm, and a lateral resolution of 6 μm. The surface roughness values of the PCL membranes fabricated with chloroform-DMF solvent were measured with the following parameters: 1.30 μs of brightness, a contrast of 3.6, objective overview of 20X, vertical resolution of 0.60 μm, and a lateral resolution of 6 μm. Figure 10 illustrates roughness values collected from the material samples produced with acetic acid and with chloroform-DMF, respectively. Notice that the highest roughness values are attained in the membrane samples developed with acetic acid. Therefore, it can be concluded that chloroform-DMF solvent shows an effective interaction with PCL and TiO_2_ nanoparticles.

## 4. Conclusions

In this study, PCL and TiO_2_ NPs composite membranes were successfully fabricated using acetic acid and chloroform-DMF solvents. Through experimental measurements of their physicochemical properties, it was found that the composite membranes elaborated with chloroform-DMF exhibit better surface homogeneity than those elaborated with acetic acid. A decrement in the degree of crystallinity is reported for the PCL reference membrane sample elaborated with chloroform, which indicates that the solvent and addition of TiO_2_ NPs influences the PCL composite membrane’s crystallization. Furthermore, XRD experimental results show a wavenumber shift in the main PCL diffraction planes for composite membranes fabricated with chloroform-DMF, which is an indication of good solubility and miscibility with PCL. Therefore, composite membranes elaborated with this solvent promote uniform macro pore structure interaction between reagents. Furthermore, the affinity, compatibility, and interaction that TiO_2_ NPs have with PCL and chloroform-DMF enhances compound properties such as strength, ductility, surface roughness, and contact angle, among others. In fact, uniaxial experimental data show that the samples manufactured with chloroform-DMF solvents exhibit higher moduli, tensile strength, and elongation values than those obtained with acetic acid solvent. The same tendency was observed during equibiaxial tests. Therefore, it can be concluded that composite membranes produced with PCL, TiO_2_ nanoparticles, and chloroform-DMF solvents in general, exhibit superior physical, chemical, and mechanical properties than those developed with PCL, TiO_2_ nanoparticles, and an acetic acid solvent. 

In accordance with the obtained results, it is evident that the developed composite material based on PCL-TiO_2_ nanoparticles with chloroform-DMF solvents broadens the practical applications of material blends based on PCL to fabricate scaffolds for engineering musculoskeletal tissue, drug delivery devices, and biodegradable plastics, to name a few. 

## Figures and Tables

**Table 1:** Description of the solutions used to produce the polymeric membranes.

Sample	Compound	Solvent	PCL (%)	TiO_2_ (M)	P25 Degussa (M)
A1	PCL	Acetic acid	10	-	-
A2-D	PCL-P25	Acetic acid	10	-	0.015
A3-D	PCL-P25	Acetic acid	10	-	0.030
A2-Sg	PCL-TiO_2_ 400 °C	Acetic acid	10	0.015	-
A3-Sg	PCL-TiO_2_ 400 °C	Acetic acid	10	0.030	-
C1	PCL	Chloroform-DMF	10	-	-
C2-D	PCL-P25	Chloroform-DMF	10	-	0.015
C3-D	PCL-P25	Chloroform-DMF	10	-	0.030
C2-Sg	PCL-TiO_2_ 400 °C	Chloroform-DMF	10	0.015	-
C3-Sg	PCL-TiO_2_ 400 °C	Chloroform-DMF	10	0.030	-

**Table 2:** Solvent Physical Properties, adapted from [16,17].

Solvent	Boiling Point (°C)	Relative Polarity	Solubility Parameter (cal^1/2^ cm^−3/2^)	PCL Solubility	Porogenic Type
Chloroform	61.2	0.259	9.3	Good	Non-Solvating
Acetic Acid	118.1	0.648	13.01	-	Solvating
DMF	62.6	0.386	11.79	Good	-

**Table 3:** Onset melting temperature ($T_{m}^{onset}$), enthalpy for melting ($\Delta H_{m}^{0}$), and degree of crystallinity ($\chi_{c}$) values retrieved from DSC.

Samples	$T_{m}^{onset}\;({}^{\circ}C)$	$\Delta H_{m}^{0}\;(J/g)$	$\chi_{c}\;(\%)$
PCL Reference	58.8	54.7	39.2
A1	56.6	74.7	53.6
C1	55.9	42.3	30.3

**Table 4:** Young modulus, maximum tensile and elongation at break mechanical properties experimentally obtained from the different membrane compounds samples when subjected to uniaxial extension tests.

Sample	Maximum Tensile Stress, MPa	Elongation at Break, m/m	Young Modulus, MPa
A1	2.36	0.82	69.91
A2-D	1.91	0.60	51.3
A3-D	2.45	0.55	85.81
A2-Sg	2.08	0.47	62.75
A3-Sg	2.79	0.39	93.54
C1	6.04	2.35	141.97
C2-D	7.47	2.46	258.77
C3-D	13.49	2.87	400.47
C2-Sg	11.67	2.37	374.77
C3-Sg	15.47	2.93	485.29

**Table 5:** Material constants used to fit the cyclic experimental data.

Sample	*μ* (MPa)	*N* (-)	*A*_1_ (MPa)	*A*_2_ (MPa)	*b* (-)	*c* (MPa)	*f* (-)	Permanent Set
A1	130	4	0	−2700	7	2.5	0.7	0.035
C1	450	40	0	0	10	15	0.6	0.001
